# Discovering Effective Connectivity in Neural Circuits: Analysis Based on Machine Learning Methodology

**DOI:** 10.3389/fninf.2021.561012

**Published:** 2021-03-16

**Authors:** Pedro Pozo-Jimenez, Javier Lucas-Romero, Jose A. Lopez-Garcia

**Affiliations:** Department of Systems Biology, University of Alcalá, Madrid, Spain

**Keywords:** AI algorithm, effective connectivity, multielectrode recordings, spinal cord circuits, machine learning, C5.0

## Abstract

As multielectrode array technology increases in popularity, accessible analytical tools become necessary. Simultaneous recordings from multiple neurons may produce huge amounts of information. Traditional tools based on classical statistics are either insufficient to analyze multiple spike trains or sophisticated and expensive in computing terms. In this communication, we put to the test the idea that AI algorithms may be useful to gather information about the effective connectivity of neurons in local nuclei at a relatively low computing cost. To this end, we decided to explore the capacity of the algorithm C5.0 to retrieve information from a large series of spike trains obtained from a simulated neuronal circuit with a known structure. Combinatory, iterative and recursive processes using C5.0 were built to examine possibilities of increasing the performance of a direct application of the algorithm. Furthermore, we tested the applicability of these processes to a reduced dataset obtained from original biological recordings with unknown connectivity. This was obtained in house from a mouse *in vitro* preparation of the spinal cord. Results show that this algorithm can retrieve neurons monosynaptically connected to the target in simulated datasets within a single run. Iterative and recursive processes can identify monosynaptic neurons and disynaptic neurons under favorable conditions. Application of these processes to the biological dataset gives clues to identify neurons monosynaptically connected to the target. We conclude that the work presented provides substantial proof of concept for the potential use of AI algorithms to the study of effective connectivity.

## Introduction

The understanding of neuronal circuits within the nervous system has become a major focus of interest in current neurobiology. The advent of novel techniques, such as those enabling the monitoring of neuronal activity across populations of neurons, is opening the door to circuit analysis. Depending on the biological preparation used, electrode matrixes may record action potentials from dozens to thousands of neurons producing huge amounts of data. Usually these recordings are obtained under blind conditions and the structural and functional relation between the recorded neurons is unknown or insufficiently described. As more laboratories use multielectrode recordings, the issue of reconstructing the effective connectivity between the different units recorded is becoming a focus of major interest in neurobiology. Effective connectivity refers to the influence that one neural system exerts over another (Friston, 2011). At the level of single neurons, it involves the analysis of temporal causality between different activations of neurons in a network (Andalibi et al., 2016).

The common approach to the study of effective connectivity is the use of inferential procedures based on statistical tools. These include methods based on cross-correlation analysis, useful only for pairs of spike trains, methods based on maximum likelihood and generalized linear models, which are sophisticated and expensive in computer terms (Masud and Borisyuk, 2011; Andalibi et al., 2016).

Here we set out to test the hypothesis that standard algorithms of machine learning or artificial intelligence could be used to analyze large datasets from electrophysiological recordings and produce relevant information on the temporal relations between the spike trains of neurons leading to a better understanding of their effective connectivity. These procedures are capable to deal with large numbers of spike trains with a relative economy of computer time.

For the present work, we have followed the lines of development suggested by the methodology CRISP-DM (Chapman et al., 2000; Shearer et al., 2000). One of the main aspects of this methodology is the modeling step. Since our priority was to gain insight on the workings of neuronal circuits we chose the C5.0 algorithm (Kuhn and Johnson, 2013) evolved from the C4.5 (Quinlan, 1993). This algorithm is based in decision trees and it can generate rules and reason for its decisions. Rules are expressed in intelligible terms for humans and define the path from the root to the leave. This later characteristic we thought could be helpful to understand the circuits under analysis although we did not focus on this issue for the present work. Furthermore, the algorithm can produce a ranking of neurons based on their relevance to the firing of the target. Some alternatives, like Artificial Neural Networks (ANN) or Deep Learning (LeCun et al., 2015), can be oriented to obtain higher successful percentages in metrics at the risk of losing the interpretability that C5.0 offers.

To achieve our goal we have used different datasets. Several of them were obtained from an artificially built circuit consisting of 80 neurons by selecting spike trains form subsets of neurons. Since the structural connectivity of the circuit was known, we used these datasets to evaluate the precision of the predictions made by the algorithm under different conditions.

An additional dataset was obtained from an electrophysiological experiment consisting of 13 well isolated neurons plus the natural target of the system, a primary afferent. This dataset was used to explore whether or not the procedures elaborated could be applied to biological problem circuits to obtain clues about effective connectivity.

Our laboratory is focusing its activity on the study of neuronal circuits located in the spinal cord, which are responsible for the generation of antidromic firing (backfiring) in primary sensory afferents. Backfiring in nociceptive afferents is related to the maintenance of inflammation leading to pain and allodynia and it is a phenomenon of physio-pathological relevance (Willis, 1999; Cervero et al., 2003). The neurons causing primary afferent depolarization and backfiring are located in the superficial laminae of the spinal cord. They are GABAergic neurons (Willis, 2006) but the basic aspects of the circuit controlling them are largely unknown. For the experiment presented here, we have used a spinal cord slice preparation form mice pups in which backfiring of primary afferents occurs spontaneously. With this preparation, we can obtain simultaneous recordings from afferents and of dorsal horn neurons to generate datasets containing the precise timing of action potentials in all these elements.

## Materials and Methods

In this section, we describe how simulated and biological data were obtained, prepared, and processed.

### Simulated Data

To obtain the simulated neural data we used a spiking cortical network model developed by Izhikevich (2006) later modified by Ito et al. (2011). We have adapted this model reducing the number of neurons and scaling down (to 5) the number of connections per neuron while keeping the ratio of inhibitory/excitatory neurons, the connectivity restrictions for inhibitory neurons as well as a fixed delay for inhibitory synapses.

Using the model, we created a **large** circuit containing 80 neurons and chose one excitatory neuron as our target. This large circuit consisted of **two unconnected** circuits of 40 neurons each (30 excitatory and 10 inhibitory). This was done to ensure availability of spike trains unrelated to the behavior of the target neuron as expected from blind biological recordings.

Synaptic parameters for excitatory neurons were defined as follows:

- The synaptic delay was a random time between 1 and 5 ms.
- The synaptic strength was a variable parameter according to spike timing dependent plasticity, as explained in the original description of the model (Izhikevich, 2006).
- The maximal synaptic strength was limited as in the original circuit. Under our conditions, at least two excitatory inputs have to occur simultaneously to cause a discharge in the postsynaptic cell, as in the original model circuit.

In the Izhikevich model, spontaneous activity of the circuit is sustained by an external excitatory input delivered at random times to a set of neurons that can be defined. By defining which neurons of the circuit receive this external input we can make the target neuron’s activity more or less predictable based on the behavior of the neurons that are directly connected to it. Thus, we defined three different **levels of uncertainty**:

- In the situation with higher degree of uncertainty every neuron in the circuit received this random external input, so that some of the spikes of the target cannot be predicted by the circuit activity.
- Preventing the target neuron from receiving this external input, we made it entirely dependent on the presynaptic units, achieving a medium degree of uncertainty.
- The lowest degree of uncertainty was obtained when we also removed the external input from neurons monosynaptically connected to our target.

For each of these scenarios, “maximal synaptic strength” and “external input strength” were adjusted to maintain the firing frequency in the target within a comparable range.

The MATLAB code used for simulations is attached as Supplementary Material (Simulation_code.m).

### Acquisition of Biological Data

Experimental data were obtained from an *in vitro* experiment using a neonate mice spinal cord slice obtained as previously described (Lucas-Romero et al., 2018). Briefly, the lumbar segments of the spinal cord were dissected from anesthetized animals (urethane 2 g/Kg i.p.) and kept in cold sucrose-substituted artificial cerebrospinal fluid (ACSF). Meninges were removed in a cold plate, and the L4 dorsal root was gently teased in order to obtain thin rootlets. Then, a single horizontal slice of 400 μm containing the dorsal horn and attached dorsal roots was sectioned (Sectioning Systems 1500, Vibratome). The slice was transferred to a recording chamber and maintained at 22 ± 1°C with oxygenated ACSF (composition in mM: NaCl 127, KCl 1.9, KH_2_PO_4_ 1.5, MgSO_4_ 1.3, CaCl_2_ 2, NaHCO_3_ 22, and glucose 10, pH 7.4).

Extracellular recordings from dorsal horn neurons were obtained with a 32-channel multielectrode array (MEA, Buzsaki 32-A32 from NeuroNexus). Recordings from dorsal rootlets were obtained by placing a single rootlet into a tight fitting glass suction electrode. Back-propagating action potentials in dorsal rootlets were recorded in AC mode. Signals from the 33 channels were processed by an Intan amplifier (RHS2000 Stimulating-Recording System, Intan Technologies).

Recordings were digitized and stored for offline analysis using hardware and software from Cambridge Electronic Designs. Spike sorting was performed with a semi-automatic procedure with the aid of principal components analysis using Spike 2 software under supervision of a trained researcher (see Supplementary Figure 1). In this way, we obtained simultaneous spike trains from a set of dorsal horn neurons and several primary afferents of which one was selected as target. The experiments recorded unperturbed spontaneous activity in absence of external stimulation (Lucas-Romero et al., 2018).

### Data Preparation

Time of occurrence of action potentials from the different spike trains were extracted, kept in separate channels and stored in CSV files for posterior analysis. The first part of this analysis consisted in the definition of different types of intervals. When a spike was detected in the target we recorded its time stamp and defined a **positive interval**. Then, we search for spikes of individual neurons in the preceding 50 ms.

The remaining temporal space was divided in 50 ms intervals and the last millisecond of the interval was considered its timestamp. These were defined as **negative intervals** (absence of spike in target). The same procedure was followed for simulated and biological data.

**FIGURE 1:**
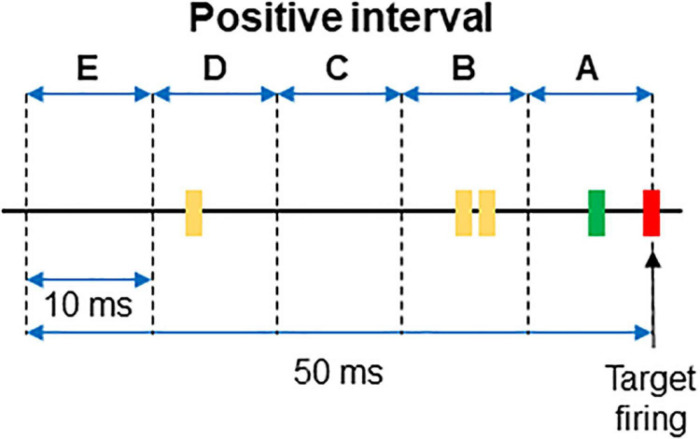
Example of a positive interval. The firing of the target unit is represented by the red line, while the spikes of two different dorsal horn neurons are plotted in green and yellow, respectively. The 50 ms interval preceding the firing of the afferent was divided in 10 ms subintervals named from A to E. The interval represented in the figure corresponds to that of the first row in Table 1.

For each interval the firing or absence of firing of the target was recorded as 1/0. Then, the firing of each neuron was categorized and labeled as A if it occurred within 10 ms from the time stamp and as B if it occurred between 10 and 20 ms from the time stamp. C, D, and E labels were applied to spikes occurring at the successive 10 ms subintervals (see Figure 1 for clarification). When no spikes occurred, the label 0 was assigned. If two spikes occurred in the same subinterval, a double code was applied. An example of categorization of several intervals is shown in Table 1.

**TABLE 1:** Defining categories.

**Time (s)**	**U1**	**U2**	**U3**	**U4**	**U5**	***R***
23.456	0	A	BBD	0	0	1
23.450	0	A	ABD	0	0	0
48.550	A	0	C	E	AB	0
103.566	0	0	0	0	0	1
109.300	0	0	0	0	0	0

*Rows represent five different intervals defined by their time stamps shown in first column. Columns U1 to U5 display the behavior of each of five dorsal horn neurons or units at each interval. The last column (R) shows the firing of the afferent. Two intervals were positive (*R* = 1, the afferent fires) and three were negative (*R* = 0, the afferent does not fire). A–E label the occurrence of spikes at the corresponding subintervals. Two identical letters represent the occurrence of two spikes in the same interval. The absence of spikes is labeled by 0. The interval casuistry is represented: firing in dorsal horn neurons and primary afferents (row 1), firing in neurons but not in afferents (rows 2 and 3), firing in afferents but not in neurons (row 4), and neither type of element fires (row 5). The first row is represented graphically in Figure 1.*

### Modeling, Validation and Tuning

For this proof of concept, we chose the algorithm C5.0 which belongs to the decision trees family. As previously stated, this model offers a traceability of decision making that *a priori* we considered important. Kuhn and Johnson (2013) discuss the main theoretical framework and characteristics of the algorithm in depth. In comparison to its predecessor C4.5 (Quinlan, 1993), the new algorithm includes upgrades such as the use of a cost-sensitive matrix or boosting.

There is no universal rule to define the percentages of cases assigned to training and validation. Here we follow previous work and Pareto’s Principle (Pareto, 1896; Dunford et al., 2014; Serrano et al., 2017) and use 80% of positives for training and 20% for validation. A larger percentage in validation may lose too much information needed for learning, but a smaller percentage may give an improper confidence because of the variability associated to a small amount of cases.

Since we have very few positives in comparison with a large number of negatives, our data sets are clearly unbalanced (Drummond and Holte, 2003; Weiss et al., 2007) and therefore our first model prototypes using all data did not fit correctly. In order to enable a proper training of the model, we used random undersampling and cost-sensitive matrix. We tested several undersampling ratios from 1:200 to 1:1 (1:200, 1:100, 1:50, 1:25, 1:10, 1:4, 1:3, 1:2, 1:1). We chose 1:4, which means that the number of negative cases was four times the number of positives. With this undersampling ratio, we obtained the best estimation of firing in the target reducing overfitting differences between training and validation in comparison to other ratios.

We also assigned a value of 3.5 for false negatives (FN) and 1 for false positives (FP) in order to build up the cost-sensitive matrix. This value was fixed after testing several values in order to weight false negatives in the cost matrix after having fixed the undersamplig ratio to 1:4.

The “trials” parameter was set to a value of 1. Again, we used several values for this variable and decided on 1 because it gives more clear results. A comparison between 1 and 5 trials is shown in results.

Following these procedures, four different sets of data can be defined as follows:

- **Complete dataset:** set of data containing all positive and all negative cases.
- **Snap data:** Subset containing all positive intervals and a proportion of negatives (1:4).
- **Training data:** Subset containing the 80% of snap data.
- **Validation data:** Subset containing the remaining (20%) of snap data.

Rules were obtained by the C5.0 algorithm from the training subset and tested in the validation, snap and complete datasets. We present and discuss the results obtained in the complete dataset, which represents the more realistic sample of the real phenomenon.

In order to increase analysis exactitude and to ensure a true randomness on election of negative intervals (Arcuri and Briand, 2011), we trained the model 30 times with different sets of negative intervals to build different snap data sets. Data partitioning in training and validation datasets was also randomized. In order to ensure replicability of results, we set a specific random seed which is disclosed in the code repository where we made available all the code used for the present study (see below).

### Metrics

In order to summarize the performance of the algorithm we used metrics extracted from the confusion matrix (Ting, 2017). For the “Results” section we used the convention presented in Table 2 for true positives (TP), true negatives (TN), false positives (FP), and false negatives (FN).

**TABLE 2:** Conventions.

**Predicted\Actual**	**0**	**1**
**0**	TN	FN
**1**	FP	TP

*TN, True Negative; TP, True Positive; FN, False Negative; FP, False Positive.*

The metrics used in this study were as follows:

- **Precision:** Proportion of Predicted Positives cases that are Real Positives (Powers, 2011).

• $$Precision=\frac{TP}{FP+TP}$$

- **Recall** or **Sensibility:** Proportion of Real Positive cases that are Predicted Positives (Powers, 2011).

• $$Recall=\frac{TP}{FN+TP}$$

- **Matthews Correlation Coefficient** (**MCC**): It is a general performance evaluation score ranging from -1 to +1 (Matthews, 1975) which takes into account the ratio of all values included in the confusion matrix, especially on unbalanced datasets (Chicco, 2017).

• $$MCC=\frac{TP^{*}TN-FP^{*}FN}{\sqrt{{\left(TP+FP\right)}^{*}{\left(TP+FN\right)}^{*}{\left(TN+FP\right)}^{*}\left(TN+FN\right)}}$$

Metrics can be obtained using all cases or subsets of cases as described in the previous section. Further, metrics can be obtained for single neurons or any desired group of neurons extracted from the neuronal sample as we describe below. To simplify description of results we use only MCC values referred to complete sets of cases to judge the predictive value of a neuron or a group of neurons over the behavior of the target.

### Workflows

With the model adjusted as discussed, we first analyzed the datasets for the neuronal samples under consideration and obtained the identification of neurons used to build rules as well as the associated metrics. By running this procedure 30 times with different seeds, we obtained an idea of the variability of our main metrics due to random election of negative cases in the training subset.

We also obtained individual metrics for each neuron training a model per neuron as feature and the target neuron as class.

In order to find subgroups of neurons with better metrics than those of individual neurons or the entire neuronal sample we tested three different procedures (Guyon and Elisseeff, 2003):

- **Combinatory:** We trained one model for each possible subgroup of neurons created from our complete neuronal sample. The number of models is given by the combination without repetition of ***n*** neurons taken in groups of ***r*** neurons, where r is a value between 1 and n.

$$numberofpossiblemodels=\sum_{r=1}^{\text{n}}\frac{\text{n}!}{\text{r}!\left(\text{n}-\text{r}\right)!}$$

Since the group with best metrics may change depending on the seed selected, we ordered all groups by decreasing MCC value and looked at the frequency with which each neuron appeared in groups within the first percentile. The group of neurons identified by this procedure was called the “relevant group.”
- **Iterative processes:** We ranked all neurons according to their individual MCC values and then trained the model with subsets of neurons so that the less significant neuron in the rank was removed at each epoch. We developed this workflow to check if the best metrics were obtained by groups formed by the neurons with best individual metrics.
- **Recursive process:** The process starts with an analysis of the complete neuronal sample and produces two groups of neurons based on **variable importance** of each neuron. Variable importance is a metric automatically generated by C5.0 which indexes the weight that a neuron has in the taking of decisions^1^. The primary group contains all neurons with variable importance greater than 0, and the secondary group contains the remaining neurons (variable importance = 0). Then, the process executes two new analyses using the neurons from the primary and secondary groups separately. This process is repeated until reaching the level of the single neuron. If a given branch of the analysis could not fit a model, this branch was stopped while the others were continued. When the model used all neurons to build rules, we manually placed neurons with the higher values of variable importance in a group and those with lower values in another group.

### Implementation of the Analysis in a Computer System

A system built in R, automatized the whole process of analysis. The code as well as a readme file containing the necessary explanations, have been made available at https://github.com/Pedrodpj92/Afferents_learning. The system reads the data from a CSV file and after questioning the user about the specific analysis to be run, it completes all the different workflows and returns the metrics obtained as well as relevant additional information. The C5.0 algorithm was obtained from https://github.com/topepo/C5.0 and used into our program.

All analysis and processes can be performed in a commercial desk top computer.

## Results

First, we used a simulated spiking cortical network model previously published, validated and used in other functional connectivity studies (Izhikevich, 2006; Ito et al., 2011). Several datasets obtained from the simulated circuit served to study how the choice of neurons selected for analysis impacted on the performance of the algorithm.

At each subsection we report results from the analysis of extracellular recordings obtained in a biological experiment. Since the structure of the biological circuit is unknown, there is no possibility to check on the validity of the predictions made. The intention of this analysis was to see whether the algorithm could fit a model to the biological data in spite of the variability associated to complex biological circuits.

### Circuits and General Metrics

**FIGURE 2:**
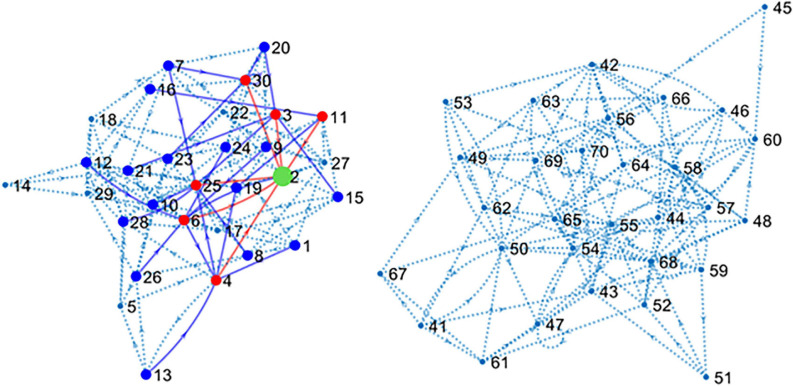
Connectivity map of the large simulated circuit. The sub-circuit in the left contains the target unit (U2 in green) and 39 connected neurons. Red dots and lines represent neurons monosynaptically connected to U2. Blue dots and lines show disynaptic connections. Dotted lines represent the remaining connections. The sub-circuit in the right contains the 40 units disconnected from U2. Inhibitory neurons have been hidden for clarity.

**The main simulated data set** was obtained by including spike trains from all the 80 neurons or units (U1–U80) that form the main circuit as explained in methods. Since the simulated circuit does not have an explicit target, we chose an excitatory neuron (U2) as target. Further, since blind electrophysiological recordings may be obtained from neurons unrelated to the target, 40 neurons (U41–U80) were disconnected from the target although connected among themselves, forming a “parallel circuit.” In this way, two independent networks formed our 80-neuron circuit (see Figure 2).

The connectivity map shown in Figure 2 presents the structure of the circuit in detail. There are six excitatory neurons involved in monosynaptic connections with the target U2. These neurons are U3, U4, U6, U11, U25, and U30. The list of excitatory neurons connected disynaptically with the reference unit is wider including 24 neurons. Some monosynaptic neurons also form disynaptic links with U2.

While keeping constant the structure of the circuit, we made different simulations changing the conditions of external excitatory random input received by the neurons of the circuit; therefore generating different conditions of uncertainty (see “Materials and Methods”). Simulations lasted for 1000 s giving rise to ∼20000 intervals.

A first run of C5.0 under conditions of low uncertainty returned large values of recall and precision leading to an MCC value of 0.84. The group of neurons used to build rules or “primary group” returned by the model was formed by seven neurons (U6, U25, U4, U3, U11, U30, U1; see Supplementary Tables 1, 2 for confusion matrix and variable importance data). Remarkably, all monosynaptic neurons were included in this group. Using 30 different seeds, we obtained a low level of variability (SEM = 0.014), supporting our undersampling and data partitioning strategies. Table 3 shows the metrics obtained for this dataset and its corresponding subsets.

**TABLE 3:** Analysis of 80-neuron circuit.

	**Direct – 1 seed**			**Mean – 30 seeds**			**SEM – 30 seeds**		
**SUBSET**	**Precision**	**Recall**	**MCC**	**Precision**	**Recall**	**MCC**	**Precision**	**Recall**	**MCC**
Complete	0.715	0.996	0.839	0.578	0.995	0.747	0.021	0.001	0.014
Snap	0.968	0.996	0.978	0.936	0.995	0.956	0.006	0.001	0.004
Training	0.971	1.000	0.982	0.941	0.999	0.961	0.005	0.000	0.003
Validation	0.955	0.982	0.960	0.915	0.981	0.933	0.009	0.003	0.007

However, as the external input was changed to increase the level of uncertainty, direct application of C5.0 returned a larger primary group including all monosynaptic neurons as well as an increasing number of irrelevant neurons. With the highest level of uncertainty, the primary group was formed by 32 neurons.

As a first conclusion from this preliminary study, it seems that a direct run of C5.0 returns primary groups which are very dependent on the level of uncertainty. A single run of C5.0 may be useful only when all or most neurons involved in a circuit are included in the data set.

**During the biological experiment**, we isolated 13 dorsal horn neurons (or units U1–13) and five primary afferents. Analysis were run with all afferents and we selected as target the afferent that produced better metrics. Neurons had a mean firing frequency of 0.9 ± 0.35 Hz (range 0.03–3.88 Hz) and their firing patterns were classed as irregular simple (10 units), irregular fast burst (2), and regular simple (1) following criteria previously reported (Lucas-Romero et al., 2018). The afferent had an irregular firing pattern with a mean firing frequency of 0.09 Hz.

After gathering a complete dataset with ∼38000 intervals, we obtain a snap data subset with 175 positive and 700 negative intervals. The confusion matrix for this dataset is shown in Supplementary Table 3. Direct application of C5.0 obtained a primary group formed by nine neurons (U09, U11, U02, U03, U05, U04, U06, U07, and U13; see Supplementary Table 4 for variable importance data). For this complete dataset, the MCC value was 0.16 (see Table 4 for metrics from all subsets). Using 30 different seeds, the SEM obtained was 0.01, suggesting again unbiased undersampling and data partitioning.

**TABLE 4:** Analysis of experimental dataset.

	**Direct – 1 seed**			**Mean – 30 seeds**			**SEM – 30 seeds**		
**SUBSET**	**Precision**	**Recall**	**MCC**	**Precision**	**Recall**	**MCC**	**Precision**	**Recall**	**MCC**
Complete	0.062	0.489	0.164	0.083	0.479	0.180	0.010	0.006	0.010
Snap	0.813	0.489	0.567	0.814	0.479	0.558	0.014	0.006	0.004
Training	0.793	0.483	0.552	0.822	0.502	0.577	0.013	0.008	0.003
Validation	0.900	0.514	0.629	0.776	0.383	0.473	0.023	0.013	0.016

### Analysis of Different Sets of Neurons

**FIGURE 3:**
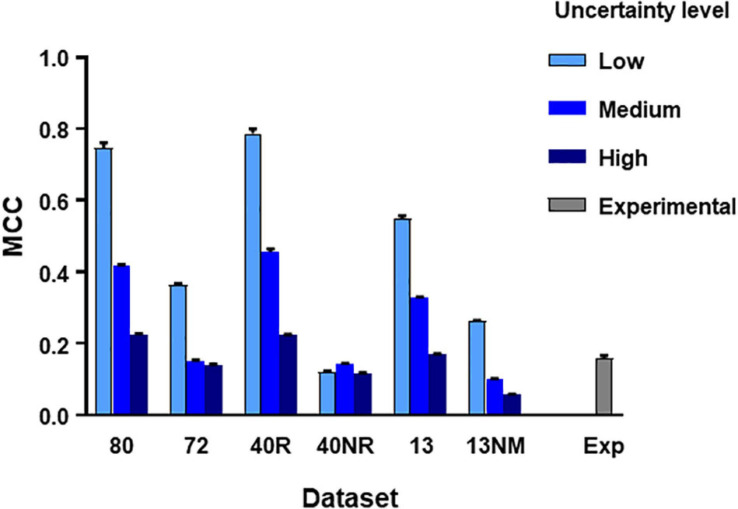
MCC values obtained from direct application of C5.0 to different datasets under different levels of uncertainty as indicated. The mean and SEM values shown were obtained after 30 runs of the algorithm using different seeds. Datasets in the horizontal axis as follows: **80,** all neurons in large simulated circuit; **72,** as before but spike trains from monosynaptic neurons removed; **40R,** medium circuit with 39 neurons connected to the target plus target itself; **40NR,** 40 neurons disconnected from target; **13,** reduced dataset from 13 neurons forming mono-, di-, or tri-synaptic contacts with U2 plus disconnected neurons; **13NM**, same as before with spike trains form monosynaptic neurons replaced by those of neurons with lower degree of connectivity; **Exp,** experimental dataset.

From the **main simulated dataset** analyzed in the previous section, we obtained different subsets by eliminating the spike trains produced by certain neurons as specified. Then we run the C5.0 30 times with different seeds to obtain mean MCC values (±SEM) and repeated the procedure under three levels of uncertainty as defined in methods. The aim of this test was to establish how the number of neurons, their degree of connectivity to the target and the level of uncertainty affected the metrics obtained. Figure 3 summarizes these results.

The subsets studied were as follows:

- A large dataset containing spike trains from all neurons except those monosynaptically connected to the target.
- A medium sized dataset containing spike trains from the 39 neurons connected to the target.
- A medium sized dataset containing spike trains from the 40 neurons not connected to the target.
- A reduced dataset containing spike trains from 13 neurons including neurons of all types and degrees of connectivity with U2.
- A reduced dataset containing spike trains from 13 neurons excluding neurons mono-synaptically connected to the target.

Results of this test lead to three basic conclusions (see Figure 3). First, the MCC value is sensitive to the level of uncertainty generated by external random input (*p* < 0.0001 in all comparisons, 2-way ANOVA, Tukey’s multiple comparisons test). Second, the MCC is sensitive to the number of neurons included in the dataset. Reduced datasets produced lower metrics. This is consistent with the previous observation since a reduction of the number of neurons included in the dataset is equivalent to increasing the level of uncertainty. The third and most important issue is that MCC is sensitive to the degree of connectivity of the neurons included in the dataset. When spike trains from monosynaptic neurons were removed from large or reduced datasets, the metrics obtained decreased significantly (*p* < 0.0001 for all comparisons, 2-way ANOVA, Tukey’s multiple comparisons test).

Mean MCC and SEM values for the **biological dataset** are included in Figure 3. These values are similar to those obtained for the simulated dataset formed by spike trains from 13 neurons containing monosynaptic neurons, under conditions of high uncertainty.

### Analysis of Individual Neurons

**FIGURE 4:**
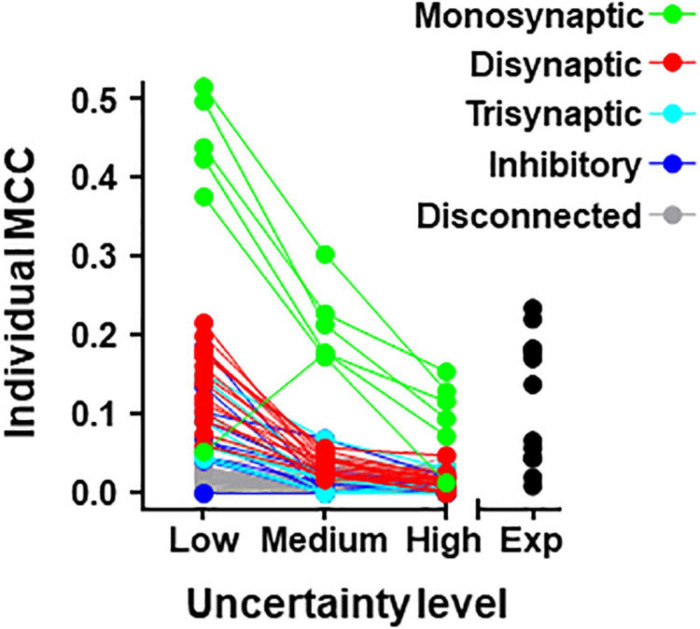
Individual MCC values for neurons contained in the 80-neuron simulated circuit under low, medium, and high levels of uncertainty. Neuron types are defined by their connectivity with the target as labeled. The MCC values for the experimental data (Exp) are included.

Individual metrics were obtained for the 80 neurons forming the **simulated circuit** under different conditions of uncertainty. Results for MCC values are shown graphically in Figure 4 (values for precision and recall are included in Supplementary Table 5). The larger MCC values for individual neurons correspond to neurons monosynaptically connected to U2. Under the most favorable conditions of uncertainty, these values were between 0.38 and 0.51. However, a monosynaptic neuron had lower values due to its low firing frequency.

For neurons belonging to the simulated circuit connected to the target, individual metrics fell considerably when the uncertainty increased. Neurons disconnected from U2 had low metrics regardless of the level of external input.

The list of individual metrics for simulated neurons includes monosynaptic neurons in the first positions.

Metrics for individual neurons were also obtained for the **biological dataset**. Detailed data resulting from this analysis are shown in Table 5 and Figure 4. The neuron with highest individual MCC was U11 (MCC = 0.232) which fells within the range of MCC values obtained by monosynaptic neurons of the simulated circuit under medium conditions of uncertainty.

**TABLE 5:** Individual metrics for experimental dataset.

**Name**	**Precision**	**Recall**	**MCC**
U11	0.350	0.157	0.232
U05	0.312	0.163	0.223
U08	0.306	0.107	0.179
U02	0.321	0.101	0.178
U09	0.122	0.253	0.170
U03	0.244	0.112	0.163
U10	0.175	0.101	0.130
U06	0.051	0.101	0.066
U04	0.022	0.230	0.057
U01	0.033	0.079	0.044
U13	0.011	0.326	0.038
U07	0.009	0.157	0.019
U12	0.000	0.000	0.000

### Analysis Using the Combinatory Process

From the data already reported it is clear that groups of neurons can have better metrics than those obtained by individual neurons. Therefore, we decided to search for a “relevant group,” that is a group formed by the smallest number of neurons that explains most of the MCC.

**FIGURE 5:**
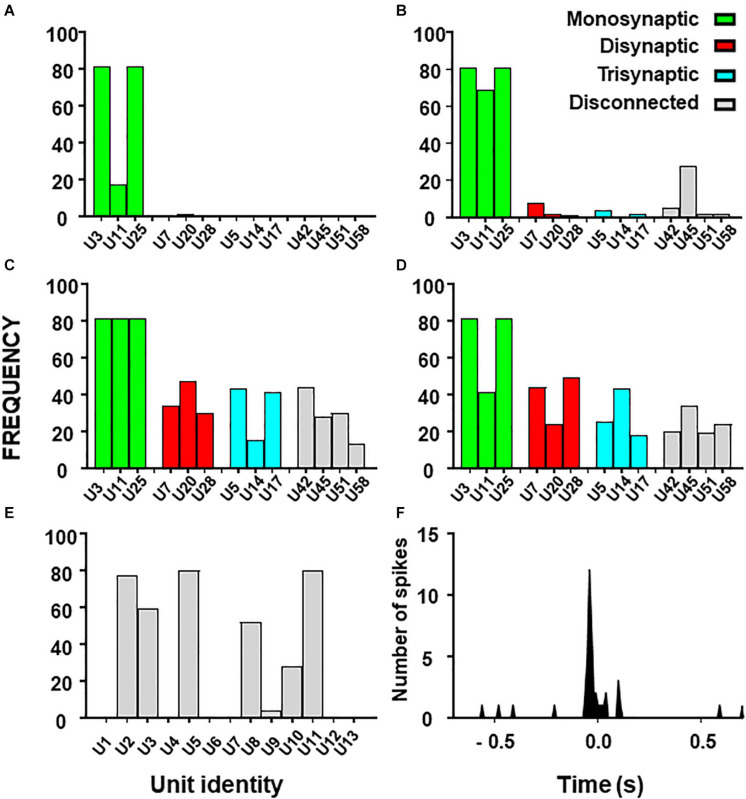
Histograms from combinatory analysis showing the frequency of appearance of each neuron in top 1% groups ordered by MCC value. Results from the 13-neuron simulated circuit containing monosynaptic neurons are shown in **(A–D)**: **(A)** low uncertainty, one decision tree; **(B)** high uncertainty, one decision tree; **(C)** as in ‘**(A)**’ with five decision trees; **(D)** as in ‘**B**’ with five decision trees. Bar colors, represent the connectivity to the target as labeled. Results obtained with the biological dataset are shown in **(E)**. All bars are in gray because their connectivity to the target is unknown. The cross-correlogram between a dorsal horn neuron (U11) and the afferent (as trigger) is shown in **(F)**. Note how the firing in the neuron tends to precede firing in the afferent.

The procedure described in methods was followed to find the relevant group using only datasets with 13 neurons from the **simulated circuit**. The total number of possible groups per analysis was 8191. Figure 5 shows the frequency with which each neuron appeared in the first 1% of groups with better metrics (values of metrics for groups are shown in Supplementary Table 6).

First, we used the dataset formed by spike trains from 13 neurons, some of them monosynaptically connected to the target. Using one decision tree as standard, the frequency histograms obtained under conditions of low and high external input discriminate clearly the monosynaptic neurons (Figures 5A,B). Then, we examined the effects of using five decision trees as shown in Figures 5C,D. Under this condition, monosynaptic neurons still had larger frequencies than others but disynaptic, trisynaptic and disconnected neurons started to appear in groups with good metrics.

Finally, we performed similar analysis using the dataset with spike trains from 13 neurons of which none was monosynaptically connected to the target. Results show that discrimination of di-synaptic neurons was poor, especially under conditions of high uncertainty (data not shown).

The outcome from these observations is that the capacity to define a relevant group by the combinatory process relays on the strength of the effective connections to the target and on the amount of uncertainty contained on the dataset. In addition, the use of a single decision tree gives optimum results.

Results for the combinatory analysis applied to the **biological dataset** are shown in Figure 5E (complete results are shown in Supplementary Table 7). The neurons more frequently appearing in groups within the top 1% of best MCC values were U11, U5, U8, U3, U2, and U10, considered as the relevant group for this dataset. The mean MCC for these top performing groups was 0.321 ± 0.0003. All neurons of the relevant group had a low firing frequency and their firing preceded that of the afferent. A representative correlogram of this kind is shown in Figure 5B. Not included in this relevant group were neurons firing at high frequencies, neurons that tended to fire after the afferent and one uncorrelated neuron. Remarkably, neurons with the best individual metrics belong to the relevant group as defined by the combinatory analysis.

### Analysis Using the Iterative Process

**FIGURE 6:**
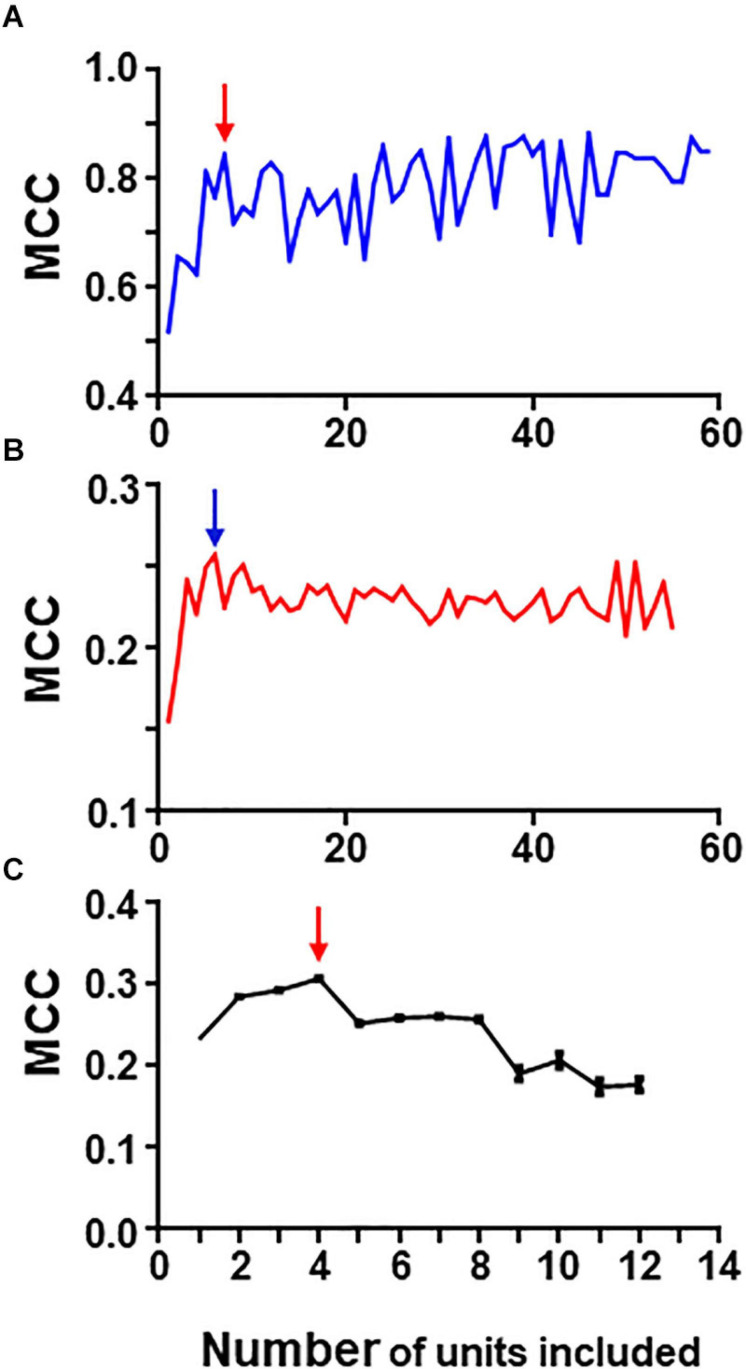
Graphs in **(A)** and **(B)** show results of iterative analysis applied to the 80 neuron circuit under conditions of low **(A)** and high **(B)** uncertainty. In both cases the critical point is reached when a group formed by the best 5–6 units remain (marked by arrows). Analysis were performed with spike trains from 59 neurons; the remaining neurons were excluded due to their low individual metrics. **(C)** Shows results for the experimental dataset (critical point marked by arrow). This analysis was run 30 times with different seeds and the data presented are mean and SEM (see text for details). In this case, the group with best metrics contained 4 units, all of them included in the relevant group.

The rational for the iterative process laid on the idea that neurons with better individual metrics, could lead to the generation of groups with the best metrics. Following the procedure explained in methods we analyzed the 80-neuron **simulated circuit** and Figure 6 summarizes the results of this analysis run under conditions of low and high levels of uncertainty (Precision and recall values are shown in Supplementary Tables 8, 9).

Under conditions of low uncertainty, we found that a small group of five neurons reaches near maximum MCC values (Figure 6A). This group was formed by all monosynaptic neurons (except U11 which has a low firing rate under this condition). Under conditions of high uncertainty, the metrics were much lower (Figure 6B); however, there is a clear point in the graph at which the MCC value starts descending. This point corresponds to the group formed by five monosynaptic neurons. In this case, U11 was included (since external input increased its firing frequency) whereas U4 was excluded.

Similarly, a “critical point” was also present in the graph collecting results from the iterative process applied to the group of 13 neurons containing spike trains from monosynaptics (not shown).

Therefore, this method seems to work well at detecting strong functional links under conditions of low and high uncertainty.

To analyze the **biological dataset** with the iterative process we applied the procedure 30 times using different seeds and report mean ± SEM values. Mean maximum MCC values fell within the range of 0.17–0.30. MCC values increased as the first neurons were discarded suggesting that those neurons introduced noise. The best MCC values were obtained when only neurons U11, U05, U02, and U08 were used to build rules followed closely by a group with only three neurons U11, U05, and U02. It is noteworthy that all these neurons were also detected by combinatory analysis as belonging to the reference group. Figure 6C shows graphically the evolution of the iterative process for this dataset. As in the case of the simulated dataset, there is a critical point corresponding to the group of four neurons with maximum MCC. Extended results using this procedure are shown in Supplementary Table 10.

### Analysis by the Recursive Process

**FIGURE 7:**
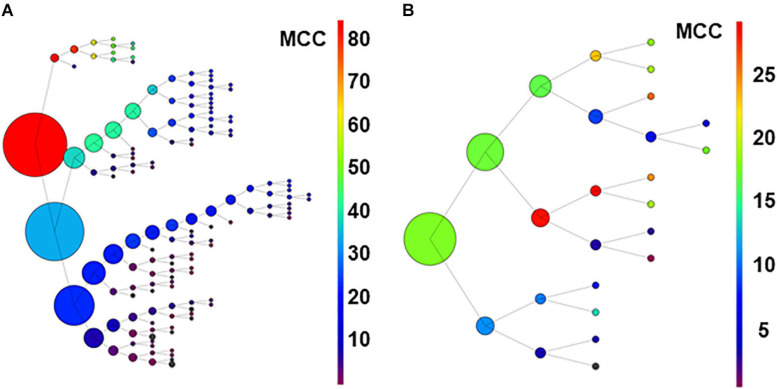
Graph in **(A)** shows results of recursive analysis applied to the 80-neuron circuit under low uncertainty. Circle size reflects the number of units contained in each group. Color scale represents the MCC value obtained according to scales. Note that the units forming best groups were separated in the top branches of the graph. This analysis required 144 executions of the algorithm. The recursive analysis applied to the experimental dataset is shown in **(B)**.

As an additional strategy to identify relevant neurons using limited computational resources, we developed a recursive process. Following the procedure described in methods for the recursive process, the 80-neuron simulated circuit was used for the first run under conditions of low (Figure 7A) and high uncertainty. Under conditions of low uncertainty, the monosynaptic neurons were selected in the upper branch (see Figure 7A). Interestingly, disynaptic neurons were included in the secondary group at the first node and later collected in the primary branch. Under conditions of high uncertainty, monosynaptic neurons were still collected at the principal branches and the smallest group with a relatively high MCC included five monosynaptic neurons but three unrelated neurons were also included (not shown; numerical data for both conditions are shown in Supplementary Tables 11, 12).

Results suggest that this procedure used under low conditions of uncertainty, may be useful to detect effective connectivity from monosynaptic and disynaptic neurons, especially when large groups of neurons are analyzed together.

For the **biological dataset**, the groups with best metrics were found in the first executions. Most important, some of the neurons forming the relevant group were included (U02, U11, U03, and U04), but others (U05 and U8) were not retrieved until several executions later, in a deep level at a secondary branch of the first analysis (see Figure 7B). Numerical data are shown in Supplementary Table 13.

## Discussion

The present results show that application of the algorithm C5.0 to the analysis of spike trains can lead to the identification of the neurons whose firing is highly relevant to the firing of a target neuron. The method proposed allows finding the information carried by spike trains of single neurons or groups of neurons, which is useful to predict the firing of the target. The method used is quantitative, revealing recall and precision data as well as combined indexes like MCC, which are important to facilitate an intuitive understanding of the predictive value of neurons. Therefore, the method produces data that can be useful to detect effective connectivity links among neurons (Friston, 2011).

We have simplified our metrics to a single value, the Matthews Correlation Coefficient or MCC (Matthews, 1975). MCC was sensitive to the degree of connectivity to the target neuron as well as to the degree of uncertainty contained in the dataset. Although the use of MCC is considered useful on a regular basis, it may be necessary to use recall, precision or other metrics under specific conditions. For example, neurons that fire spontaneously at a high frequency may obtain a considerable MCC with a high recall but little precision. Therefore, although the systematic use of MCC is useful, it requires supervision.

The experiments performed on the simulated dataset, demonstrate that a single run of C5.0 may detect all the neurons monosynaptically connected to the target under favorable conditions of uncertainty, even in complex datasets including large numbers of spike trains. The degree of uncertainty depends on a series of factors. As a general rule, the greater the number of spikes in the target neuron that cannot be explained in terms of spikes in the other neurons, the greater the uncertainty. Datasets from real electrophysiological experiments may have relatively high levels of uncertainty due mainly, but not exclusively, to the existence of relevant inputs that are hidden to the recording electrodes and therefore, excluded from the dataset. Further, some of the recorded spike trains may be engaged in processes unrelated to the target neuron adding noise to the datasets. Under conditions of medium or high levels of uncertainty, a single run of C5.0 is likely to give good clues on effective connectivity since the primary groups formed contain neurons closely related to the target although they may contain other less relevant neurons.

As a first conclusion, the present experiments indicate that the algorithm is useful to detect groups of neurons that condition the firing of the target; however, the validity of results depends critically on the level of uncertainty. Since the level of uncertainty of real electrophysiological datasets is likely to be considerable, we have explored other processes that may improve the usability of the algorithm.

First, we obtained individual MCC values for each neuron. The simulated circuit allowed us to realize that neurons monosynaptically connected to the target had the greater individual MCC values. Quantitative differences in MCC values of neurons with different degrees of connectivity to the target were considerable under conditions of low and medium levels of uncertainty but they blurred under high uncertainty. Although this process may be insufficient when sampling only a few neurons from a large biological circuit, it is possible to envisage ways of analysis of individual metrics using clustering tools to refine the prediction of monosynaptic connectivity even under high uncertainty conditions.

The results of individual metrics obtained by U11 of the simulated dataset give an additional clue about the true meaning of the information captured by the algorithm. This neuron received similar metrics to other monosynaptic neurons except for its low MCC under conditions of low uncertainty. As explained in the “Results” section, this was because U11 fired few action potentials in the absence of external input and therefore its influence on the target fell down. Although its structural connectivity was identical, its effective connectivity decreased due to low levels of activity.

Another useful contribution of single neuron analysis was to demonstrate that small groups of neurons could obtain higher metrics than single neurons. This is the reason that pushed us to search for a “relevant group,” that is, the smallest group of neurons that can explain most of the MCC.

To this aim, we designed the combinatory process. This process is time consuming due to the large number of groups that require analysis. Having access to large computer facilities should solve this problem. However, we wanted to perform all our analysis on a basic desktop available at any laboratory, therefore we performed it in reduced simulated datasets containing only 13 neurons, the size of our biological dataset.

This analysis showed very good results in the simulated datasets used. Monosynaptic neurons were clearly isolated from the rest regardless of the level of uncertainty. However, replacement of monosynaptic neurons showed that combinatory analysis does not identify disynaptic, trisynaptic or disconnected neurons with sufficient clarity. This is an important observation and it may suggest that the sensitivity of this combinatory process is limited to a single synapse.

In addition, we used the combinatory analysis to demonstrate that the use of a single decision tree leads to better results than the use of five decision trees because poorly related neurons may be collected as relevant.

Although the combinatory process may be useful for the analysis of small datasets, it is not adequate for large datasets unless a fast computer facility is at hand. For this reason, we developed an iterative process that produces graphs containing the result of eliminating one by one the neurons with worst MCC values as explained. In the graphs obtained, it is possible to identify a critical point for which the MCC value reaches a maximum with a relatively small number of neurons. For the 80-neuron simulated dataset, a group of neurons monosynaptically connected to the target obtained maximum MCC values. Of most interest is the fact that this process has been proved useful under conditions of high uncertainty. Tested in a 13-neuron dataset with monosynaptic neurons, results were similar. On these grounds, we propose that the iterative processes combined with data from individual neurons may be a good procedure to obtain a relevant group of neurons without the need to run expensive combinatory processes.

The recursive analysis may provide useful information as well. When tested in a large dataset with low level of uncertainty, the recursive analysis returned a group of monosynaptic neurons in a first branch and a group of disynaptic neurons in a secondary branch. This is our first observation indicating that the algorithm may be used to identify second order neurons under certain circumstances. Further research will be required in this direction to refine procedures that increase the sensitivity of the analytical processes.

The biological dataset analyzed in the “Results” section was obtained from a set of 13 neurons recorded in the dorsal horn of the spinal cord plus a primary afferent, which is the natural target of the circuit. The circuit regulating backfiring of primary afferents is unknown and likely to contain a number of relevant differences with the simulated circuit. However, it is remarkable that the metrics obtained in the different analysis performed with the biological dataset fell well within the limits of variability established for the simulated datasets. This suggests that the algorithm and the different processes developed may be still valid when analyzing biological circuits of unknown structure.

Using the algorithm we have obtained interesting practical clues. For example we have found that a direct application of C5.0 may provide a rapid indication about the potential interest of a given experiment so as to decide whether it is worth continuing the analysis. Furthermore, the low metrics obtained with the biological dataset indicates that the sample of recorded neurons explains only a part of the behavior of the afferent and suggests that the biological circuit contains a number of neurons considerably larger than the set of neurons actually recorded.

Looking at the individual metrics, there are at least seven neurons with MCC values considerably high and compatible with the status of monosynaptic connection to the output neuron. The correlogram shown for U11 in Figure 7 is also compatible with a monosynaptic contact. At least five or six of those neurons are included in the relevant group obtained by the combinatory analysis. Finally, four of those neurons are detected by the iterative analysis as the major contributors to the firing in the afferent. The data clearly indicate that our sample included six neurons with a strong effective connectivity to the output neuron. Some or all of them could be monosynaptically connected to the afferent. From these data, we can speculate that the afferent may receive a large number of monosynaptic inputs from different neurons and that several of them are required to coincide in time to activate the afferent. One first practical output of these observations is that recordings of larger numbers of neurons will be required to draw precise effective connectivity maps to characterize the circuits involved.

We believe that the experiments reported constitute a valid proof of concept to reinforce the potential use of C5.0 algorithm to the analysis of spike trains and effective connectivity in neuronal circuits. In addition, refinement of processes may facilitate the building of effective connectivity maps for neuronal circuits. A simple strategy for refinement would be to combine individual or recursive processes with combinatory analysis. Other alternative strategies would include the analysis of the rules created to take decisions or use of different algorithms that may produce classifications that are more accurate. A more complex strategy involves a long-term process of interaction between the use of biological and modeled datasets until models reflect true biological traits of the studied circuit.

## Data Availability Statement

The datasets presented in this study can be found in online repositories. The names of the repository/repositories and accession number(s) can be found in the article/Supplementary Material.

## Ethics Statement

The experimental protocols performed on animals were approved by the University of Alcalá Ethics Committee and covered by a license issued by the Govern of the Community of Madrid (PROEX 018/16).

## Author Contributions

PP-J adapted and developed the software and the different processes used. JL-R obtained biological and simulated spike trains. JL-G coordinated the work and wrote the original manuscript. All authors conceived this work, revised and contributed to the final version of the manuscript.

## Conflict of Interest

The authors declare that the research was conducted in the absence of any commercial or financial relationships that could be construed as a potential conflict of interest.

## Footnotes

1 https://topepo.github.io/C5.0/reference/C5imp.html
