# Supplementary material for: Discovering Effective Connectivity in Neural Circuits: Analysis Based on Machine Learning Methodology
Source: Front Neuroinform. 2021 Mar 16;15:561012. doi: 10.3389/fninf.2021.561012 (PMC8007904; doi:10.3389/fninf.2021.561012)
Supplement: Supplementary file 2 [file Data_Sheet_2.docx]

clear

% spnet.m: Spiking network with axonal conduction delays and STDP

% Created by Eugene M.Izhikevich. February 3, 2004

% Modified to allow arbitrary delay distributions. April 16,2008

%

% Modified by Javier Lucas-Romero 2020 for the paper:

%'título'

% Allowing different uncertainty levels

% We created three situations with increasing uncertainty in which

% the output/target neuron activity can be differentialy predicted

rand('seed',1); %The seed will determine the circuit architecture

Uncertainty=1; %Set this value in 1, 2 or 3 for low, medium or high uncertainty

Output_neuron=2; %Neuron considered as the circuit output

simulation_length=1000; %Time in seconds

M=5; % number of synapses per neuron

D=5; % maximal conduction delay

% excitatory neurons % inhibitory neurons % total number

Ne=30; Ni=10; N=Ne+Ni;

a=[0.02*ones(Ne,1); 0.1*ones(Ni,1)];

d=[ 8*ones(Ne,1); 2*ones(Ni,1)];

if Uncertainty==1;

sm=15; % maximal synaptic strength

Random_external_input=15;

elseif Uncertainty==2;

sm=10; % maximal synaptic strength

Random_external_input=15;

elseif Uncertainty==3;

sm=10; % maximal synaptic strength

Random_external_input=10;

else

display('Error')

return

end

simulation_length=simulation_length+350; %The first 350 seconds while the simulation is stabilizing will be ignored

delays = cell(N,D);

for i=1:Ne

p=randperm(N);

post(i,:)=p(1:M);

for j=1:M

delays{i, ceil(D*rand)}(end+1) = j; % Assign random exc delays

end;

end;

for i=Ne+1:N

p=randperm(Ne);

post(i,:)=p(1:M);

delays{i,1}=1:M; % all inh delays are 1 ms.

end;

s=[6*ones(Ne,M);-5*ones(Ni,M)]; % synaptic weights

sd=zeros(N,M); % their derivatives

% Make links at postsynaptic targets to the presynaptic weights

pre = cell(N,1);

aux = cell(N,1);

for i=1:Ne

for j=1:D

for k=1:length(delays{i,j})

pre{post(i, delays{i, j}(k))}(end+1) = N*(delays{i, j}(k)-1)+i;

aux{post(i, delays{i, j}(k))}(end+1) = N*(D-1-j)+i; % takes into account delay

end;

end;

end;

presynaptic_excit={}; %cell with excitatory neurons presynaptic to each excitatory unit

for loop=1:Ne

[r c]=find(post==loop);

r=sort(r);

r=r';

presynaptic_excit(loop,:)={r};

end

for loop=1:Ne

presynaptic_excit(loop,:)= {presynaptic_excit{loop,:}(presynaptic_excit{loop,:}<=Ne)};

end

post_excit={}; %cell with excitatory postsynaptic targets of excitatory units

for loop=1:Ne

to_delete=post(loop,:);

remaining_u=to_delete(to_delete<=Ne);

post_excit(loop,1)={remaining_u};

end

STDP = zeros(N,1001+D);

v = -65*ones(N,1); % initial values

u = 0.2.*v; % initial values

firings=[-D 0]; % spike timings

firings_saved=[];

bar=waitbar(0,'Simulating');

for sec=1:simulation_length % simulation length in sec

waitbar(sec/simulation_length);

for t=1:1000 % simulation of 1 sec

I=zeros(N,1);

I(ceil(N*rand))=Random_external_input; % external input

if Uncertainty<=2;

I(Output_neuron,1)=0; %In the level 2 of uncertinty the output neuron does not receive external input

if Uncertainty==1; %In the level 1 of uncertinty the presynaptic units do not receive external input

for loop=1:length(presynaptic_excit{Output_neuron,1})

I(presynaptic_excit{Output_neuron,1}(loop),1)=0;

end

end

end

fired = find(v>=30); % indices of fired neurons

v(fired)=-65;

u(fired)=u(fired)+d(fired);

STDP(fired,t+D)=0.1;

for k=1:length(fired)

sd(pre{fired(k)})=sd(pre{fired(k)})+STDP(N*t+aux{fired(k)});

end;

firings=[firings;t*ones(length(fired),1),fired];

k=size(firings,1);

while firings(k,1)>t-D

del=delays{firings(k,2),t-firings(k,1)+1};

ind = post(firings(k,2),del);

I(ind)=I(ind)+s(firings(k,2), del)';

sd(firings(k,2),del)=sd(firings(k,2),del)-1.2*STDP(ind,t+D)';

k=k-1;

end;

v=v+0.5*((0.04*v+5).*v+140-u+I); % for numerical

v=v+0.5*((0.04*v+5).*v+140-u+I); % stability time

u=u+a.*(0.2*v-u); % step is 0.5 ms

STDP(:,t+D+1)=0.95*STDP(:,t+D); % tau = 20 ms

end;

firings2=[firings(:,1)+1000*(sec-1),firings(:,2)];

firings_saved = [firings_saved;firings2];

% plot(firings(:,1),firings(:,2),'.'); %Uncomment to visualize the simulation second by second

% axis([0 1000 0 N]); drawnow;

STDP(:,1:D+1)=STDP(:,1001:1001+D);

ind = find(firings(:,1) > 1001-D);

firings=[-D 0;firings(ind,1)-1000,firings(ind,2)];

s(1:Ne,:)=max(0,min(sm,0.01+s(1:Ne,:)+sd(1:Ne,:)));

sd=0.9*sd;

end;

close(bar);

firings=firings_saved;

cond=firings(:,1)>350000; %Skip the first 350 s

firings3=firings(:,1);

firings4=firings(:,2);

firings=[firings3(cond),firings4(cond)];

firings(:,1)=firings(:,1)-firings(1,1);

figure

plot(firings(:,1),firings(:,2),'.');

axis_param=[0,max(firings(:,1)),0,N+1];

axis(axis_param);

firings_output=sum(firings(:,2)==Output_neuron); %Count of spikes in the output neuron

firings_presynaptic=[]; %Count of spikes in the monosynaptically connected units

for loop=1:length(presynaptic_excit{Output_neuron,1})

firings_presynaptic=[firings_presynaptic;presynaptic_excit{Output_neuron,1}(loop) sum(firings(:,2)==presynaptic_excit{Output_neuron,1}(loop))];

end

display(firings_output)

display(firings_presynaptic)

%Saving configurations parameters

parameters=cell2table({Ne,Ni,M,D,sm,Random_external_input,Output_neuron,simulation_length},'VariableNames',{'Neu_Ex','Neu_In','Syn_per_Neu','Max_Delay','Max_Strength','Basal_Input','Output_neuron','seconds'});

%Code to export simulation output in csv file

x=(1:N);

count = zeros(N,1);

for k = 1:N

count(k) = sum(firings(:,2)==x(k));

end

rows=max(count);

columns=N;

output_matrix=NaN(rows,columns);

times=firings(:,1);

for n=1:N

cond=firings(:,2)==n;

vector_times=times(cond);

output_matrix(1:length(vector_times),n)=vector_times;

end

output_matrix=output_matrix.*0.001; %output times in seconds

C2=cell(1,columns);

for n=1:columns

C2{1,n}=['U',num2str(n)];

end

C=num2cell(output_matrix);

T=cell2table(C,'VariableNames',C2(1,:));

writetable(T,'simulation_output.csv')
