# Supplementary figures and images for: Discovering Effective Connectivity in Neural Circuits: Analysis Based on Machine Learning Methodology
Source: Front Neuroinform. 2021 Mar 16;15:561012. doi: 10.3389/fninf.2021.561012 (PMC8007904; doi:10.3389/fninf.2021.561012)

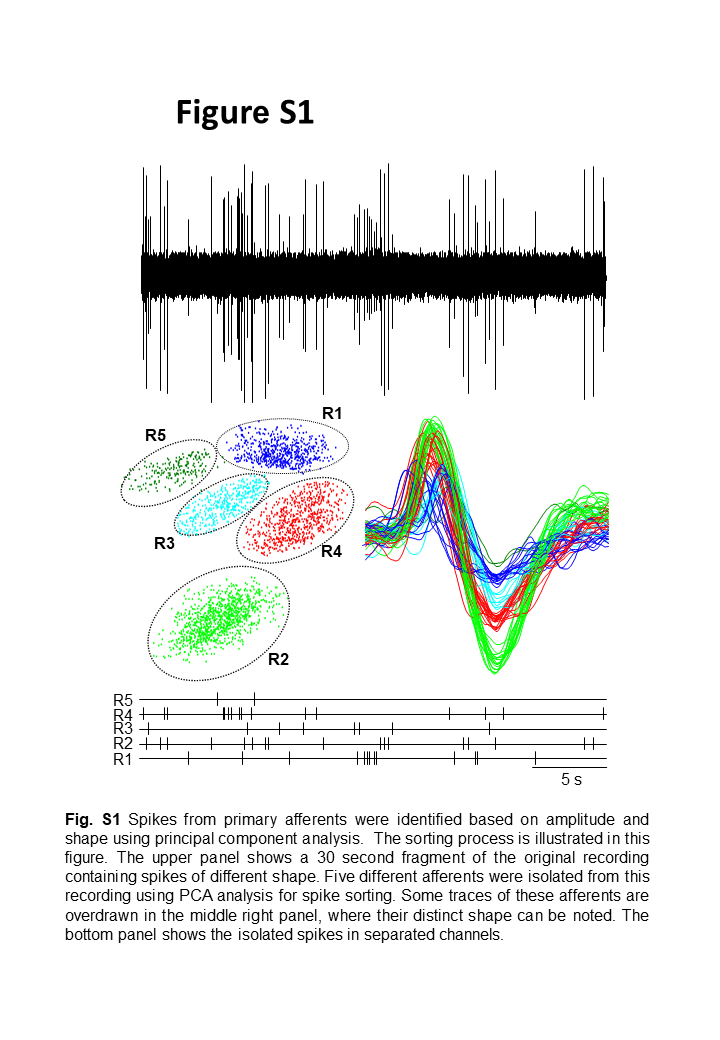

Supplement: Supplementary file 3 [file Image_1.tif]
